# A Virological and Phylogenetic Analysis of the Emergence of New Clades of Respiratory Syncytial Virus

**DOI:** 10.1038/s41598-017-12001-6

**Published:** 2017-09-25

**Authors:** Farah Elawar, Cameron D. Griffiths, Daniel Zhu, Leanne M. Bilawchuk, Lionel D. Jensen, Lydia Forss, Julian Tang, Bart Hazes, Steven J. Drews, David J. Marchant

**Affiliations:** 1 Li Ka Shing Institute of Virology, Department of Medical Microbiology and Immunology, University of Alberta, Edmonton, Alberta Canada; 2 Clinical Microbiology, University Hospitals of Leicester NHS Trust, Leicester Royal Infirmary, Leicester, UK; 3 Department of Infection, Inflammation and Immunity, University of Leicester, Leicester, UK; 4 Alberta Provincial Laboratory for Public Health, University of Alberta, Edmonton, AB Canada; 5 Department of Laboratory Medicine and Pathology, University of Alberta, Edmonton, Alberta Canada

## Abstract

The significant burden of Respiratory Syncytial Virus (RSV) in pediatric and elderly populations is well recognized. However, questions remain about transmission and evolution of RSV in the community, between seasons, and the role played by viral genetics in viral replication. Therefore, we integrated next generation sequencing, patient viral load, and viral replication analysis with surveillance of RSV to initiate a better understanding of viral adaptation in communities. RSV type-A and B infections were most closely related to RSV sequences from the USA and Asia, respectfully. The sample titres between RSV types-A and B were not significantly different. However, when the patient sample titre was compared to the phylogenetics of RSV, emergent clades were identified that we termed High Titre (HiT) clades of RSV. In conclusion, the correlation between patient viral load and replication kinetics of RSV patient isolates in culture indicated that viral genetics may determine virus replicative ability within patients. There was evolution or introduction of high-titre RSV type-A and B infections that seeded HiT clades in the subsequent year. Therefore, virological analysis of RSV isolates in conjunction with RSV phylogenetics may be a tool for predicting new clades of RSV in impending seasons.

## Introduction

During any particular season 10–30% of all specimens collected for respiratory virus testing in Northern Alberta, Canada will be positive for respiratory syncytial virus (RSV)^1^. It is one of the greatest causes of pediatric hospital admissions worldwide^2,3^, and it is a significant health problem in the elderly^4^. Despite this burden of disease, there is only one prophylactic treatment called palivizumab, and the only licensed therapeutic treatment for RSV infection, ribavirin, is rarely used. There are numerous RSV therapeutics and vaccines in clinical trials and new promising strategies are reported each year^1^. However, since RSV is a rapidly evolving RNA virus, established surveillance of circulating viral strains will be necessary to detect the emergence of mutants escaping therapeutics and vaccination once they are available^1^. Thus, understanding RSV transmission in the community could predict the most pertinent times to administer prophylaxis, prepare community health services for patient treatment, and inform vaccine preparation (once an efficacious RSV vaccine has been developed).

Clinical and population epidemiological studies of RSV infections in patients and cohorts have formed an understanding of viral shedding in the patient, and how it relates to the disease course of RSV. A recent study has comprehensively demonstrated the potential for viable RSV to transmit via aerosols in a hospital setting^5^, which may also be applicable to community settings, such as households and nurseries/kindergartens. This contrasts with RSV being exclusively transmitted via direct contact or through fomite intermediates. The predominant method of measuring RSV viral load in the upper respiratory tract is by quantitative RT-PCR (qRT-PCR) of nasopharyngeal (NP) samples^6–12^. The period of shedding of RSV lasts 3 to 10 days in infants^12^ and adults^6^, which is a significantly longer period of shedding compared to influenza^6^. Studies of natural infection in pediatric cases and experimental inoculation of healthy adults with RSV have revealed a close association of RSV qRT-PCR viral load with disease course^6–10^. A recent study showed using qRT-PCR measurement that RSV viral loads were associated with hospitalization of infants with RSV infection^12^. When the aerosol transmission of RSV is considered, the patient NP viral load is not only a correlate of disease severity, but also a potential correlate of increased transmissibility. This information might inform health care investment and prepare for hospitalizations if virulent strain emergence of RSV can be predicted by community surveillance studies.

Despite the genomic atlas of sequencing information on the circulating strains of RSV in the NCBI database, there are still many questions surrounding the transmission, virulence, and clade evolution of RSV types-A and B. There are reports that RSV type-A is more virulent than RSV type-B in the hospital setting^13,14^, and some that suggest the two RSV types are equally as virulent^1^. During our study period, we observed a prevalence of RSV type-B infections. Furthermore, there is the question of how the two RSV types remain genetically distinct, since RSV types-A and B share 95% sequence identity and RSV mutates rapidly in the community. One could presume that the two viruses would converge over time towards the most fit form. An explanation is that the alternating seasons of predominance of RSV type-A or RSV type-B in the community may help to maintain the two types as genetically distinct; undergoing alternating periods of predominance in pediatric populations^1^. In addition, we propose that RSV types-A and B may have remained genetically distinct by infecting slightly different niches within the population.

Next generation sequencing has provided an overview of the strains and clades of RSV types-A and B that are circulating worldwide^15^. Studies that utilized NGS of community RSV samples have provided a high-resolution understanding of the phylogenetic adaptation of RSV that occurs in a community^16–18^. This has led to two models for the emergence of virulent strains of RSV. Since RSV mutates during a patients’ RSV infection, this could result in the emergence of “novel” strains with greater virulence over existing clades within the community^18^. Alternatively, a population that lacks immunity to a new foreign strain of RSV would promote transmission of the newly introduced strain^15,19^. However, RSV is known to either evade or suppress B cell memory in humans^20^ and, as a result, we are repeatedly infected by the same strains. We provide support here for the postulate that RSV clade formation is driven by the highest titre of infection in the host. Previously published NGS studies on RSV have not explored the relationship between patient viral load and the phylogenetic adaptation of RSV that occurs from season to season. Therefore, questions remain about the nature and significance of what drives RSV clade formation.

We have reported on disease severity and respiratory virus load in pediatric out-patients previously^11^. In this cross-sectional pilot study, we surveyed RSV isolates collected in a variety of settings from patients with a broad range of ages. We included a systematic review and meta-analysis of RSV cases worldwide to compare as a reference with our observations of RSV type-B prevalence in infants. We observed new clades of RSV type-A and type-B emerge in our sample set in just two seasons (2014–2016); there was introduction of new strains into the community in 2014 from international sources but there is also support for local evolution of RSV type-A and B clades over seasons in the local population. We identified a correlation between the RSV isolate replication kinetics and the autologous qRT-PCR viral titre in the patient samples. These experimental observations validated the viral load observations in this heterogeneous set of patient samples. The phylogenetic structures of patient RSVs were correlated to the qRT-PCR viral titres in the patients’ samples to identify the clades of RSV with the highest viral loads, which we termed High Titre (HiT) clades. We found that RSV infections with the highest viral loads in the 2014–2015 season likely seeded the RSV type-A and B HiT clades in the subsequent 2015–2016 season. Studies such as this one provide support for RSV clade establishment in the community based on the highest viral titre and form the basis of surveillance programs to predict RSV virulence. They also serve as models for vaccine efficacy testing and surveillance.

## Materials and Methods

### Antibodies

Anti-goat IgG H&L conjugated to β-Galactosidase; cat. no. ab136712 (Abcam), and Anti-RSV polyclonal; cat. no. B65860G (Meridian Life Science, USA).

### Luminex NxTAG respiratory virus panel determination

Residual clinical specimens were taken for this study. The clinical laboratory supports many practice settings and so there is no standard protocol for guiding clinicians as to when to take a respiratory specimen. Specimens are collected for patients who present with a wide variety of respiratory symptoms, not just influenza-like illness, and precise onset of illness is not known. Patient information for this study was taken from laboratory requisitions. Specimens collected were a variety of nasopharyngeal specimens including swabs and aspirates. Swabbing strategies vary in different clinical settings and institutions may include well-collected true nasopharyngeal swabs, mid-turbinate specimens, nasal swabs and even swabs additionally applied to the pharynx or applied to the pharynx alone.

All respiratory specimens were routinely tested for influenza A and influenza B using a real-time reverse transcriptase-real time polymerase chain reaction (RT-PCR) assay as previously described^11,21^; if found negative, they were tested by the NxTAG® Respiratory Virus Panel (RVP, Luminex, Austin, TX, USA) for other common respiratory pathogens including, RSV types-A and B^22^. Clinical respiratory samples from Alberta residents received at the PROVLab between 2014 and 2016 that tested positive for RSV were eligible for the study.

### Determination of viral load in patient samples

Viral RNA was extracted from patient NP samples using Qiagen QIAamp Viral RNA kit. Reverse-transcription and amplification are both performed in the same reaction using Quanta qScript XLT One-Step RT-qPCR ToughMix, with no ROX reference dye (95134-100). Taqman primer/probes (FAM) were purchased from Life Technologies these were designed to target the RSV nucleocapsid: RSV type-A (all 5′-3′: Forward - GCT CTT AGC AAA GTC AAG TTG AAT GA, Reverse - GCC ACA TAA CTT ATT GAT GTG TTT CTG, Probe - ACA CTC AAC AAA GAT CAA CTT CTG TCA TCC AGC) and RSV type-B (all 5′-3′: Forward - GAT GGC TCT TAG CAA AGT CAA GTT AA, Reverse - TGT CAA TAT TAT CTC CTG TAC TAC GTT GAA, Probe - TGA TAC ATT AAA TAA GGA TCA GCT GCT GTC ATC CA)^11^. The RNA was run on CFX96^TM^ Real-Time system, C1000 Touch Thermal Cyler by Bio-Rad. Parameters were set for an initial run at 50 °C for 12:00 minutes (1), 95 °C for 1:00 minute (2), 95 °C for 7 seconds (3), 60 °C for 45 seconds (4), a plate read (5), then a repeat from 3, 45 times. Quantification of FFU/mL from C_T_ value for each virus is derived from a standard curve of a prototypical laboratory strain RSV type A2 virus of known infectivity run in parallel with all samples during each PCR run. C_T_ thresholds were computed for each run by the Bio-Rad CFX Manage software (Version 3.1) based on a control curve that was run in parallel with each plate run.

### Culture of viral isolates from patient swabs

To culture viral isolates, we diluted the original NP sample in 1 mL of DMEM containing 10% FBS and penicillin/streptomycin. This mixture was added to subconfluent HEp-2 cells in a 6-well tray (~1.2 × 10^6^) and incubated at 37 °C in a humidified incubator and 5% CO_2_. Media was changed with 1 mL DMEM containing 10% FBS and penicillin/streptomycin after four hours, and the virus was left to propagate for 96 hours. After 96 hours, the media was harvested and stored in liquid nitrogen.

### Immunostaining for RSV infection in tissue culture

RSV infected cells were detected by adaptation of a protocol that was described previously^21,22^. Confluent HEp-2 cells were inoculated with a 10-fold serial dilution of RSV containing media for 3 to 4 hours at 37 °C in a humidified incubator with 5% CO_2_. Cells were washed and incubated overnight in fresh growth media. After 24 to 48 hours, cells were fixed and permeabilized with 1 part methanol and 1 part acetone (v/v), blocked with PBS containing 5% FBS, and stained with goat polyclonal anti-RSV antibody (Meridian, USA). The cells were washed and probed with a rabbit anti-goat secondary antibody conjugated to β-galactosidase. Infected cells were stained blue by addition of X-Gal substrate (5-bromo-4-chloro-3-indoyl-β-galactopyranoside) in PBS containing 3 mM potassium ferricyanide, 3 mM potassium ferrocyanide, and 1 mM magnesium chloride. RSV foci of infection were counted and measured by light microscopy using the EVOS FL Auto Cell Imaging System (Invitrogen). Foci of infection were detected automatically by a color segmentation program on the EVOS imaging system and the sizes were recorded as µm^2^.

### Library preparation and whole virus genome sequencing

Viral RNA was extracted from NP samples using the Qiagen QIAamp Viral RNA kit. Of the 130 RSV isolates received from PROVlab, 22 RSV type-A and 22 RSV type-B were randomly selected for sequencing due to limited resources. Our criteria for choosing isolates to sequence was: an equal representation of RSV type-A and RSV type-B as well as equal representation of viruses with ‘low’ titres under 1.0 × 10^5^ FFU/mL and ‘high’ titres over 1.0 × 10^5^ FFU/mL. The data ranged from titres of 1.0 × 10^2^–1.0 × 10^8^ FFU/mL, we stratified the data to obtain natural breaks in the dataset, and used random isolates from these groups as a representation. Sequencing libraries of RSV isolates were prepared by poly-A capture of mRNA with oligo d(T) 25 beads. High-Capacity cDNA RT kit prepared the cDNA first strand. The second strand was prepared using the large fragment of DNA polymerase 1-Klenow. The cDNA was purified using the Qiagen MinElute PCR purification spin kit and prepared for sequencing using the Illumina Nextera XT DNA library prep kit for tagmentation. Further cleanup was conducted by the MagJet cleanup protocol. Sample concentration was measured using the Qubit 3.0 Fluorometer (Life Technologies) and purity was assessed using a bioanalyzer at the University of Alberta’s Molecular Biology Service Unit (MBSU). The samples were then sent to the MBSU to be sequenced on a MiSeq platform.

### Phylogenetic tree building

RSV genome sequences obtained from the MiSeq sequencer were uploaded into Geneious software^23^. Geneious was used for initial alignment of sequenced genomes to matched reference strains (Supplementary Table 3) that were downloaded from NCBI. Once isolates had a well-defined consensus sequence, ends were trimmed and the consensus sequence was used for phylogeny.

We inferred Maximum likelihood (ML) for all the trees in this study. Our alignment method used Multiple Sequence Comparison by Log-Expectation (MUSCLE) software. Alignments were created with full genome sequences from all isolates, sequence coverage varied for some isolates however, we adjusted parameters in our alignment software to accommodate low coverage of sequences in the whole RSV genome. Phylogenetic trees were constructed using both a neighbor joining method and RAxML software^24^ depending on the bootstrap value calculated. Bootstrap values were set to 1,000 replicates and evaluated confidence. The out-group for the RSV type-A phylogenetic tree was a RSV type-B strain we obtained from GenBank (www.ncbi.lm.nih.gov). The RSV type-B tree had an RSV type-A out-group to root the tree. Very low coverage sequenced isolates were excluded if they could not align with the rest of the sequenced high coverage isolates. Low coverage may be a result from low original viral load or RNA loss during the preparation steps.

### Systematic review and meta-analysis

We followed the Preferred Reporting Items for Systematic reviews and Meta-Analyses (PRISMA) criteria for the design of systematic review and meta-analysis in this study^25^. Systematic review of existing studies in PubMed that detected RSV by subtype were included in the analysis. The PubMed search was conducted with the terms “RSV” and “subtype” as well as “RSV” and “subgroup” that returned 166 hits and 321 hits respectively. All papers that studied the detection of RSV types-A and B in a clinical or community setting were included. The primary exclusion criteria were: RSV detected in subjects without differentiation of RSV subtype, molecular methods were not used to detect RSV subtypes, or patients were not stratified into a group of less than one year of age. All the authors agreed to the inclusion and exclusion of papers in the systematic review.

### Statistical analysis

To test distribution of the collective patient viral load data we conducted normality tests on the data sets. D’Agostino & Pearson, Shapiro-Wik, and a Kolmogorov-Smirnov normality test were statistically significant for normality. We therefore concluded our data had a normal distribution and we could use parametric tests when testing for differences. The *t* test was used for comparison of paired normally distributed data sets. Comparison of multiple groups was conducted by one-way ANOVA with Tukey’s test for significance. The Mann Whitney *U* test was used for data not normally distributed. The Fisher exact test was used for categorical data. Statistical analyses were performed using GraphPad Prism 7 software, all tests were two tailed with a 0.05 level of significance. Means are reported ± standard deviation.

### Ethical approval

All activities and experimental protocols using human samples were conducted under the ethical approval and oversight of the human research ethics board at the University of Alberta; project number Pro00048549. All experiments were performed in accordance with relevant guidelines and regulations: Identities of patients were blinded to the researchers by anonymized sample identifiers. All patients receiving medical treatment for their symptoms provided consent to their caregiver for the initial NP swab. Informed consent for the viral testing of the swab and downstream processing of the virus was given by the patient or their families.

## Results

We conducted a survey of RSV in a heterogenous set of patient nasopharyngeal (NP) samples from Alberta, Canada to better understand the spread of RSV in the community. Our fundamental question was whether we could identify correlations between the viral load in patient NP samples, patient age, RSV replication kinetics, and phylogenetic data. Nasopharyngeal specimens were from patients who were tested at the discretion of a health care worker in a broad range of health care settings for respiratory symptoms, which include community emergency room visits, in hospital settings or outpatient care, from patients across Northern Alberta, Canada. The majority of samples were from the capital city of Alberta, which is Edmonton. Samples however came from a variety of hospitals across the city and cities hundreds of kilometers away. Specimens are routinely sent from these settings to the central Alberta Public Health Laboratories (PROVLab) for determination of viral infection by respiratory virus panel (RVP) on a Luminex NxTAG multiplex platform. RSV positive specimens generally account for 10–30 percent of all specimens collected during a 12 month period^1^.

For the period from 2014–2016 in Northern Alberta, RSV positivity rates varied from <1% in low circulation periods to peak positivity rates of 38%, 12% and 37% positivity for the 2014, 2015 and 2016 seasons, respectively. Median RSV type-A and RSV type-B positivity over this period was 6.5%. The proportion of detections of RSV type-B (approx. 60%) was slightly more prevalent than RSV type-A (approx. 40%) for the total period (Table 1). Co-detections of RSV type-A and RSV type-B were rare (<1%).

**Table 1:** RSV A/B infections September 1, 2014–March 31, 2016.

RSV by RVP*	3701	100%
RSV Type A	1585	42.8%
RSV Type B	2108	57%
Mixed RSV A with RSV B	8	0.2%

*xTAG® Respiratory virus panel, Luminex Corporation.

**Figure 1:**
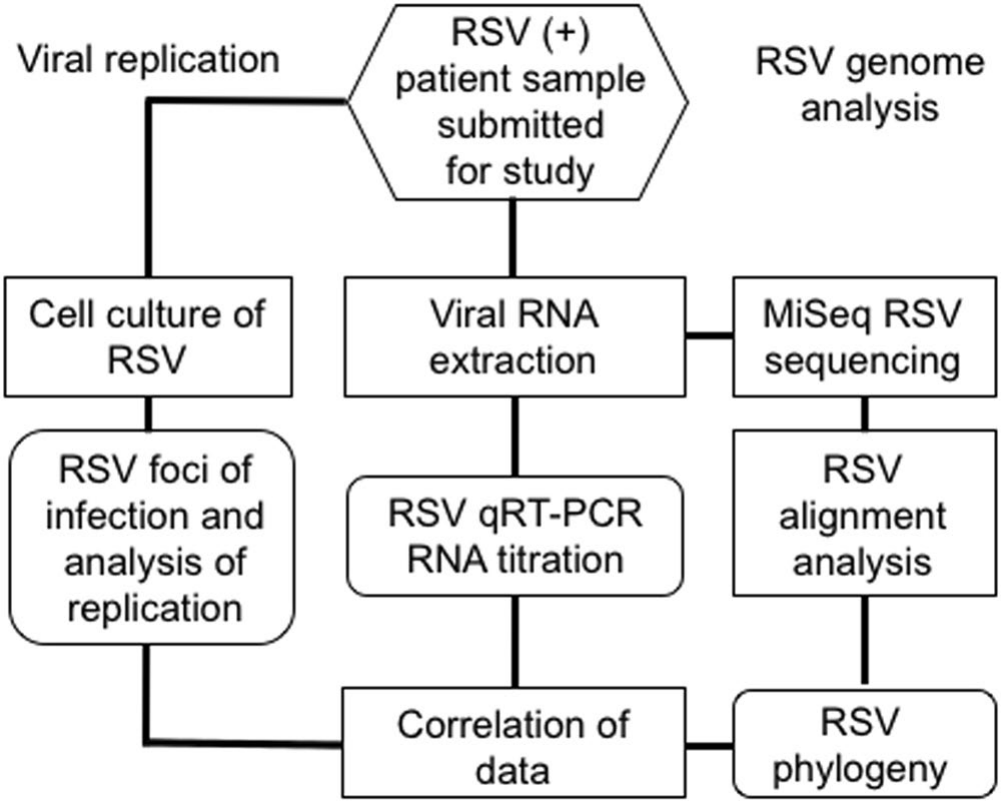
Work flow of RSV isolate culture, sample titre and sequencing from patient nasopharyngeal (NP) samples. Patient NP samples that were determined to be positive for single RSV type-A or type-B infections on a NxTAG RVP Luminex panel at the Provincial Health Laboratories (PROVLab) in Edmonton, Alberta, Canada were submitted for study. Aliquots of each sample were either propagated in immortalized HEp-2 cells or RNA extracted for RSV RNA titre determination and RSV genome sequencing analysis.

To obtain a cross-sectional population snapshot of RSV infections circulating in the community, a subset (n = 130, Supplementary Table 1) of the PROVLab RSV infected samples were randomly pulled over two RSV seasons (2014–2016). From the entire set of specimens sent to PROVLab a technician took a random subset from each month in an RSV season. Each sample was assigned an alphabetical identifier for blinding to the investigators before submission for further work-up. In the interests of simplicity, samples that were positive for more than one virus were excluded. After anonymization, the samples were submitted for titre determination by limiting dilution in cultured cells and qRT-PCR, whole genome sequencing, and culture analysis of RSV foci of infection (Fig. 1).

### There was a broad spectrum of RSV titre in patient samples

Others have reported that viral load determined by qRT-PCR is representative of viral load in the patient and is associated with the severity of patient symptoms^8,9^. Therefore the viral loads for each of the RSV type-A and type-B positive NP samples in this study were determined by quantitative RT-PCR (qRT-PCR) against a viral RNA copy standard using the method reported previously^9,11,26^. However, this analysis could not take into account how long after onset of illness the specimen was collected. When using qRT-PCR to measure viral load from patient samples, levels of viral RNA may be influenced by variations in sample collection and transport^12^. To control for this, we monitored transcript levels of a host-derived housekeeping gene, hypoxanthine phosphoribosyl transferase (HPRT) in the samples to determine whether viral load correlated with host cellular material present in the samples. We found no correlation between viral load and coincident patient cellular material (Supplementary Figure 1). We found no correlation between HPRT and any of the other metrics used to determine *in vitro* viral fitness of RSV in patient samples presented throughout this study. Therefore we do not consider normalisation of patient samples by HPRT a valid control.

The viral titres were therefore normalized to the sample universal transport media volume. The qRT-PCR viral loads in patients varied from 8.00 × 10^0^ RNA copies per mL to 3.80 × 10^8^ RNA copies/mL (Table 2). The highest titre isolates were outliers from RSV type-A infected patients with sample identifiers M and S in January 2015, with 3.80 × 10^8^ and 2.00 × 10^8^ RNA copies/mL respectively. Despite a positive RVP result, after at least 3 attempts, RSV could not be detected by qRT-PCR in 3 of the samples between 2014–2016 due to being below the threshold level of detection of our qRT-PCR assay or being degraded following initial RVP testing. Taking into account the mean titres in all of the age groups, we found that the titres of RSV type-A (2.75 × 10^7^ RNA copies/mL ± 8.99 × 10^7^ RNA copies/mL, n = 55) and RSV type-B (4.15 × 10^6^ RNA copies/mL ± 1.19 × 10^7^ RNA copies/mL, n = 52) in the NP samples did not differ significantly (p = 0.75).

**Table 2:** Summary of RSV RNA titres in Patient Samples.

RSV type-A			
**2014–2015**	<1	1–5	>25
Mean Titre (RNA copies/mL)	5.50 × 10^7^	3.36 × 10^7^	1.99 × 10^6^
Log (RNA copies/mL)	5.97	5.70	5.93
Male %	33	33	100
N	9	6	5
**2015–2016**			
Mean Titre (RNA copies/mL)	9.20 × 10^5^	3.58 × 10^5^	5.02 × 10^5^
Log (RNA copies/mL)	5.26	4.55	5.09
Male %	55	60	57
N	11	10	14
**Summary**			
Mean Titre (RNA copies/mL)	2.20 × 10^7^	1.28 × 10^7^	9.28 × 10^5^
Log (RNA copies/mL)	5.49	4.98	5.17
Male %	45	50	68
N	20	16	19
**RSV type-B**			
**2014–2015**	<1	1–5	>35
Mean Titre (RNA copies/mL)	5.74 × 10^6^	1.05 × 10^6^	9.83 × 10^6^
Log (RNA copies/mL)	5.32	4.52	5.70
Male %	70	70	40
N	10	10	5
**2015–2016**			
Mean Titre (RNA copies/mL)	1.32 × 10^6^	1.33 × 10^6^	3.04 × 10^5^
Log (RNA copies/mL)	5.37	5.40	4.53
Male %	36	63	0
N	11	11	4
**Summary**			
Mean Titre (RNA copies/mL)	3.42 × 10^6^	1.26 × 10^6^	5.59 × 10^6^
Log (RNA copies/mL)	5.35	5.00	5.18
Male %	53	67	22
N	21	21	9

### Infectious foci of RSV infection were compared to the RSV genome titre in each sample

**Figure 2:**
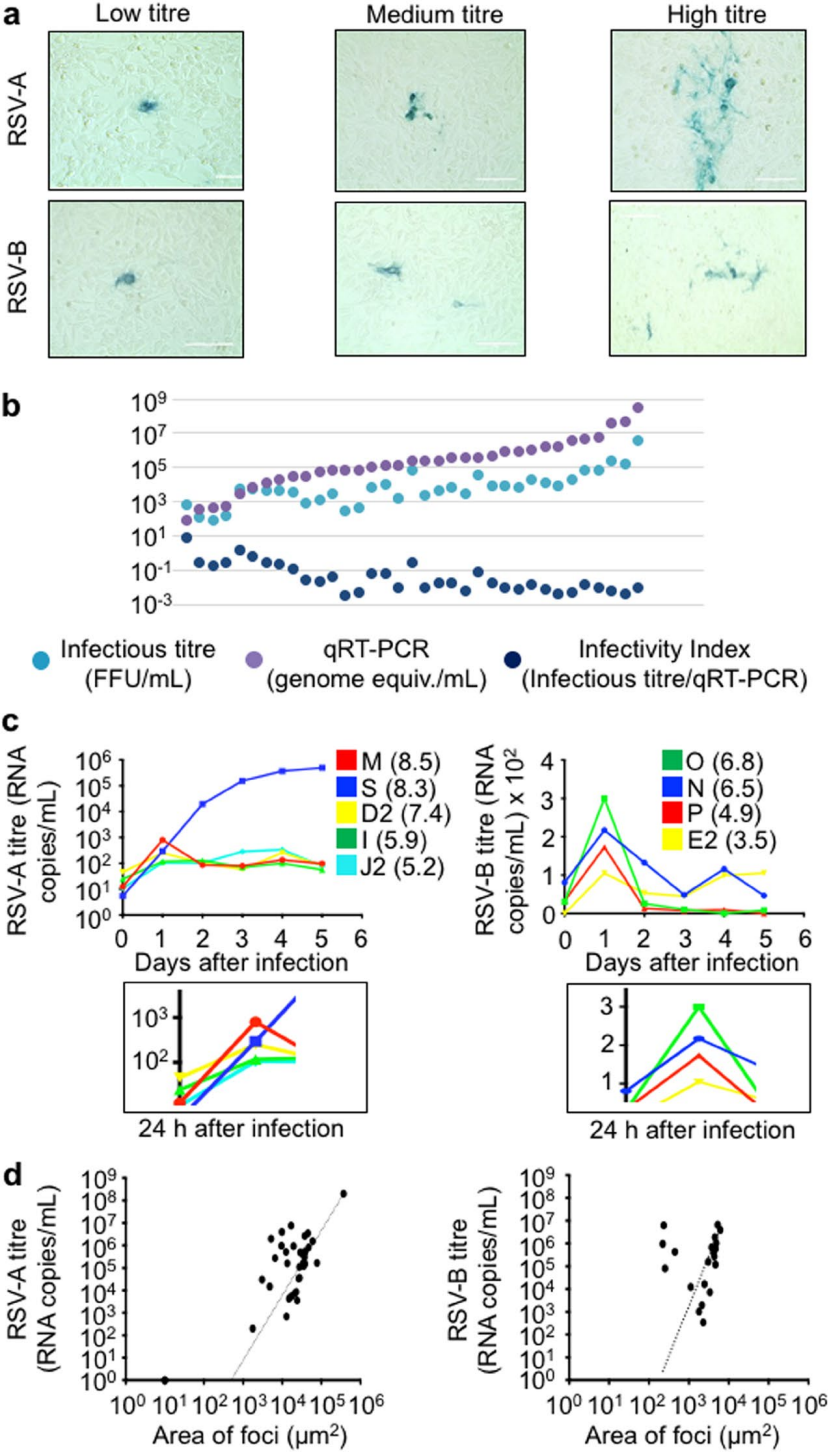
Correlation of RSV isolate replication kinetics with patient sample titre. (**a**) Immortalized HEp-2 cells were inoculated with patient nasopharyngeal (NP) samples that were determined to be RSV type-A or type-B positive by the NxTAG RVP Luminex panel at PROVLab, Alberta, Canada. The HEp-2 cells were fixed and immunostained for RSV infection two days later. The morphology and size of the plaques were analyzed using automated color segmentation software on a digital phase contrast microscope. There was a range of sizes of foci of cell culture infection among the samples. (**b**) Comparison of RSV patient sample titres were determined by qRT-PCR and the number of focus forming units per mL. Infectivity index was determined by the dividing the infectious titre (FFU/mL) by the genome equivalents/mL (by qRT-PCR). (**c**) A replication time course over 5 days of RSV type-A and type-B isolates in immortalized cells. HEp-2 cells were inoculated by equal inputs of genomic equivalents as determined by qRT-PCR of two high titre, medium titre and low titre isolates of RSV type-A and RSV type-B to determine whether there was a difference in replication kinetics of isolates of different titres in patient samples. The number in parentheses beside the sample identifier indicates the RSV titre in the original patient’s sample. Inset below: viral titres 24 hrs post infection. (**d**), RSV type-A and B titres were determined by qRT-PCR and plotted versus the area of foci of infection after 48 hours on HEp-2 cells. A line of best fit is shown for RSV type-A and type-B.

RSV from each original patient NP sample was used to infect HEp-2 cells in tissue culture in a limiting dilution series. After 48 hours of infection, the cells were fixed, immunostained for RSV antigen, and the number of infection foci were counted (Fig. 2a). RSV foci of infection were immunostained because plaque-clearings in cell monolayers were not evident with RSV infection. The number of RSV foci of infection in culture was then compared to the RSV RNA sample titre that was determined by qRT-PCR (Fig. 2b). We did this to determine whether qRT-PCR titre measurement reflected the infectious titre of RSV in the patient NP samples. In most cases, the qRT-PCR (RNA copies/mL) RSV genome equivalents were higher than the foci of infection in culture, but overall the pattern of qRT-PCR compared to the foci of infection were similar, consistent with previous reports^8,9^. We also calculated an infectivity index that was determined by dividing the infectious titre (FFU/mL) by the number of RSV genome equivalents in the samples as measured by qRT-PCR (RNA copies/mL). Despite the range of viral loads in the samples, the infectivity indices of the samples remained similar. Interestingly, there was a trend toward more infectious (higher infectivity index) virus with decreasing qRT-PCR titre in the patient samples (Fig. 2b).

### The RSV patient isolate growth rates reflect the viral titre in the patient sample

The size of the foci of infection was associated with the patient sample titre (Fig. 2a). We therefore postulated that some of the differences we observed in viral loads were characteristics inherent to the virus, rather than heterogeneity of sampling. In this case, we would expect that the RSV replication kinetics in cell culture would be associated with the viral qRT-PCR titre in each sample. To test this a time course experiment was conducted where the inputs of medium and high titre isolates were equalized based on qRT-PCR infectivity equivalents. RSV from each patient NP sample was used to infect HEp-2 cells in tissue culture, and the media was harvested after 96 hours. This first passage of RSV from the samples is relatively free of host-derived factors when compared to virus from the original patient sample. After equalizing the viral titres in the first passage samples, HEp-2 cells were inoculated with the virus isolates, and the conditioned infected media in the time courses were harvested daily from the infected cells. The level of progeny virus produced was measured by quantitative qRT-PCR (Fig. 2c). The undulating pattern of virus production at time points after 24 h was reminiscent of a defective interfering virus particle pattern of infection that occurs during time courses of viral replication, in general^27^. In summary, we found that the highest titre RSV type-A and type-B isolates demonstrated the greatest number of genomic equivalents released into the conditoned culture supernatant 24 h after incolulation (Fig. 2c). Although the mid titre isolates of RSV type-A could not be resolved, there was general agreement of RSV type-B isolate titres with the rate of replication in cell culture at the 24 h time point.

### The size of RSV foci of infection were correlated to the RSV RNA titre in patient NP samples

From the observations reported above, we postulated that RSV replication kinetics and viral load in the patient were virus encoded, rather than an artifact of sampling or extrinsic host-derived factors. Therefore, we predicted that the higher titre isolates would also form larger foci of infection in cell culture when compared to lower titre isolates (Fig. 2a). Linear regression correlated the RSV RNA titre to the area of the viral foci in patient samples. In these experiments, an aliquot of the patient NP sample was plated on HEp-2 cells, immunostained for RSV infection 24 h later, and the areas of infectious foci (µm^2^) in the sample were plotted versus the patient’s viral RNA titre (Fig. 2d). A positive correlation between the size of the foci of infection and the viral RNA titre from RSV type-A (p < 0.001; r^2^ = 0.9974) and type-B (p < 0.001; r^2^ = 0.1969) patient samples was observed. There was a trend toward larger RSV type-A (3.30 × 10^3^ µm^2^ ± 5.95 × 10^3^ µm^2^) plaque sizes compared to type-B (2.4 × 10^3^ µm^2^ ± 2.00 × 10^3^ µm^2^) though these were not significantly different when compared by a two tailed t test (p = 0.21). Some plaque morphologies were compared between RSV type-A and RSV-type-B, and differences were noted between the types (data not shown) that were consistent with what has been reported previously^28^. Therefore we had improved confidence that the viral loads we were detecting in patient NP samples were representative of the viral fitness of the virus that was isolated from the patient.

### RSV titre in stratified age groups

**Figure 3:**
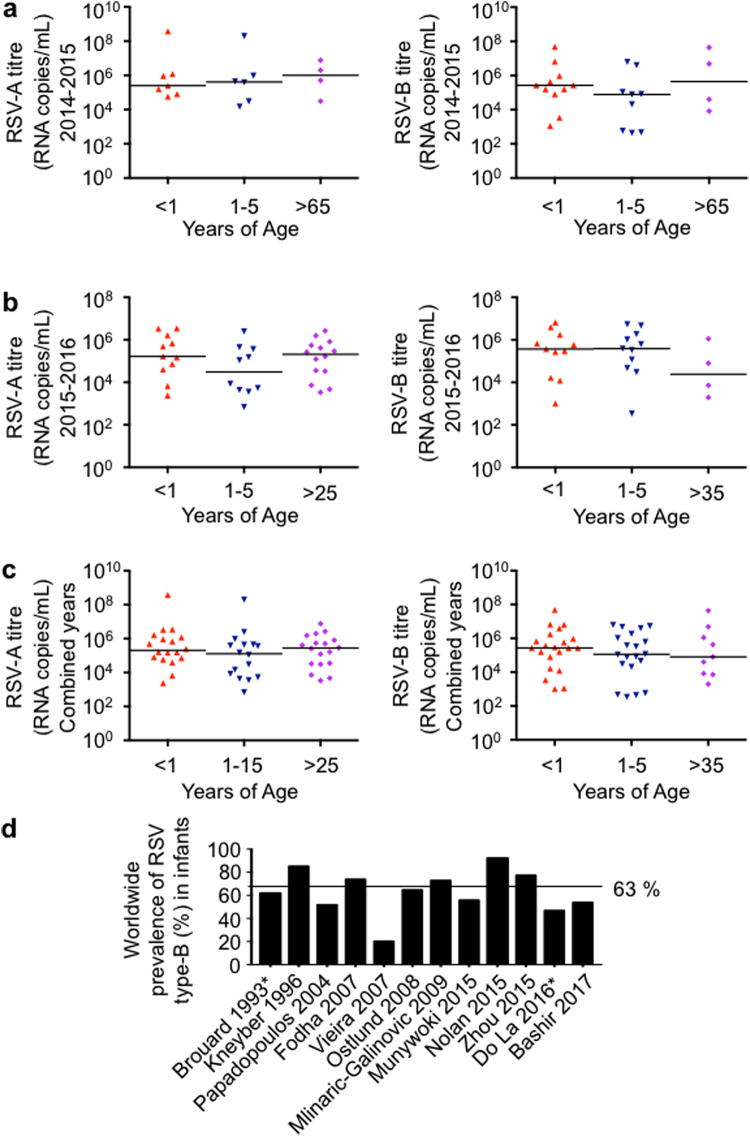
Viral titre versus stratified age groups during the 2014–2015 and 2015–2016 seasons in Alberta Canada. (**a**) 2014–2015 RSV type-A and B viral loads versus age stratification. RNA was extracted from patient nasopharyngeal samples, and RSV was quantified by qRT-PCR. The RSV RNA copies per mL were determined by comparison to a known viral quantitative RT-PCR standard. (**b**) 2015–2016 RSV type-A and RSV type-B viral loads versus age stratification. (**c**) Combined 2014–2016 RSV type-A and B viral loads versus age stratification. Horizontal lines represent the median of the data. (**d**) Summary of a systematic meta-analysis of clinical studies examining RSV type-B prevalence in those under 1 year of age. *Only stratified those less than 2 years of age or no age stratification reported. The dotted line indicates the summary mean of RSV type-B prevalence among the studies.

The RSV samples were stratified into groups based on patient age. Age groups were as follows: less than one year of age, 1 to 5 years of age, and greater than 65 years of age (Fig. 3). In the following 2015–2016 season we noticed samples belonging to adults under 65 so the stratification shifted to include them as well: less than one year of age, 1 to 5 years of age, and greater than 25 years of age (Fig. 3).

Plotting the viral loads versus age group revealed a distinct pattern of higher prevalence RSV type-B in individuals less than one year of age during the 2014–2015 season (Fig. 3a). Furthermore, we randomly sampled more RSV type-B cases in this group during the 2014–2015 season. We therefore postulated that infants less than one year of age may be more susceptible to infection by RSV type-B in some seasons.

### Systematic review and meta-analysis of infant hospitalization due to RSV type-A and B

To cross reference our results with others, we postulated that a systematic review and meta-analysis of previous studies may support a higher prevalence of RSV type-B in those less than 1 year of age. A survey of 12 clinical studies cited in PubMed that met our inclusion criteria and passed exclusion criteria^26,29–39^ (Supplementary Table 2) supported the postulated predominance of RSV type-B in infants less than one year of age (Fig. 3d). In a combined total of 823 RSV-positive infants, 217 were infected with RSV type-A and 590 were infected with RSV type-B, which is an RSV type-B predominance of 63% in those less than one year of age. The mean hospitalization times in the meta-analysis of infants less than 1 year of age did not differ between the two RSV types which were 7.6 days for RSV type-A infection and 8.17 days for RSV type-B infection (p = 0.75). We were unable to determine the prevalence of RSV type-A and B in other stratified age groups by meta analysis due to the paucity of this information in publications cited in PubMed. In summary, these data provide evidence suggesting that specific age groups may be more susceptible to certain types of RSV during certain seasons.

### Sequencing RSV genomes revealed clades of RSV type-A and RSV type-B in Alberta

**Figure 4:**
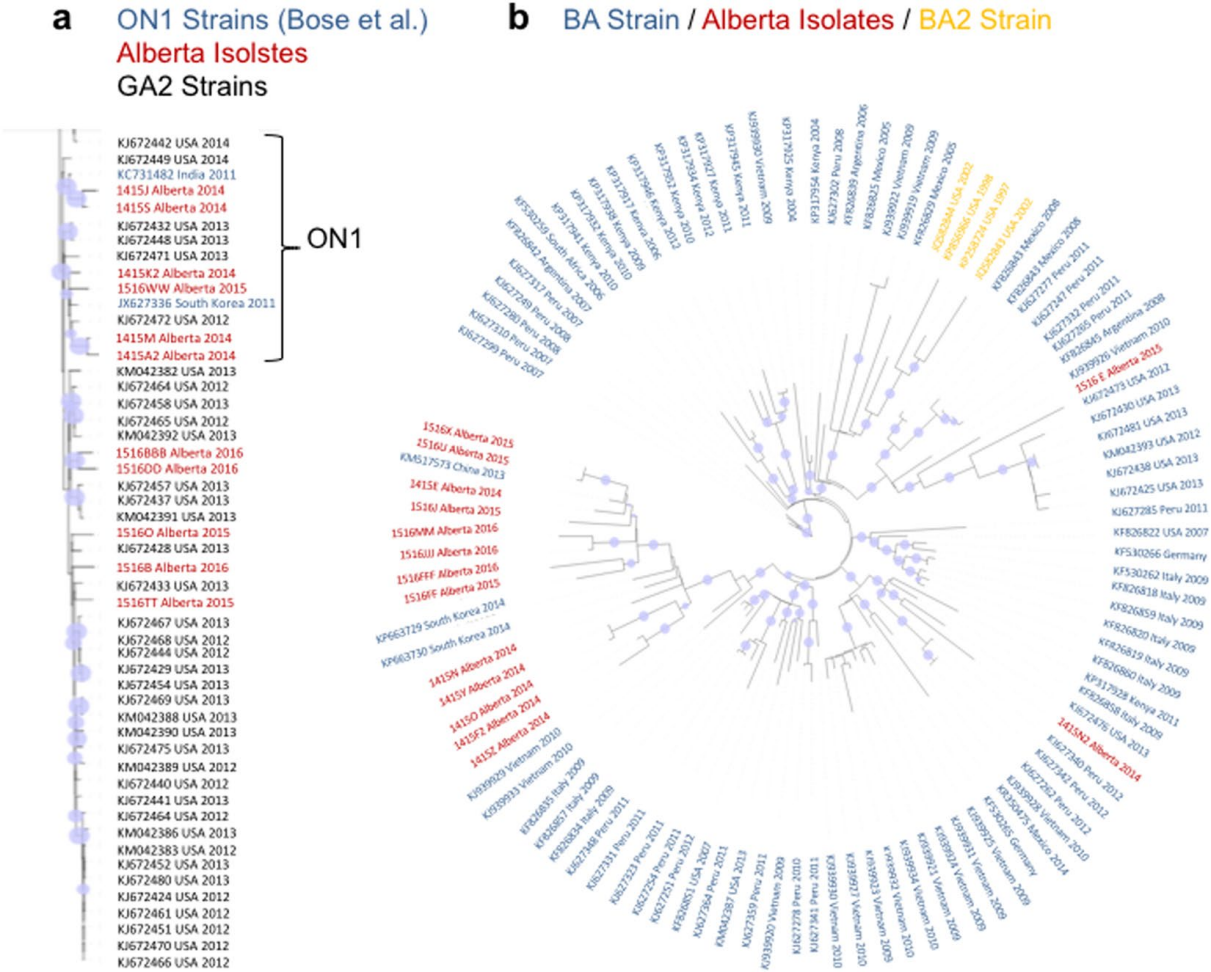
Comparison of RSV phylogenetics of Alberta, Canada patient NP sample titres during the 2014–2015 and 2015–2016 seasons to RSV strains that have been published in the NCBI database. The phylogenetic tree was created using RAxML software from an original MUSCLE alignment of the complete genomes. Reference strains previously published or identified from known genotypes obtained from the NCBI genome database are indicated above the trees. Tree topology were supported by bootstrap analysis. The blue circles indicate 100% bootstrap confidence. The tree was visualized using a Portable Network Graphics file that was downloaded into Geneious software. (**a**) A subset of a complete unrooted tree from all RSV type-A genotypes published in NCBI along with Alberta strains obtained for this study. The entire tree was too large to visualize our isolates in the context of the NCBI database, therefore we zoomed into the clade containing Alberta isolates (shown in Red). (**b**) The unrooted tree from a complete download of all RSV type-B genotypes published in NCBI. Alberta isolates are indicated in red. All isolates were post 2004 therefore the tree only shows the BA strains and the BA2 clade superseding it.

Patient sample viral loads and the rates of replication of patient isolates from the samples in culture appeared to be a virus-intrinsic product. Therefore, we employed next generation sequencing to sequence the RSV genomes in the samples. RNA was isolated by poly-dT beads from patient NP samples, libraries were prepared by Tagmentation and then sequenced on an Illumina MiSeq platform. Viruses with at least 50% sequence coverage were rendered into phylogenetic trees using maximum likelyhood to observe the differences between the viral genomes in Alberta and the genomes of RSV submitted from studies worlwide (Fig. 4). We observed sporadic groupings of RSV type-A that were interspersed among sequences from the USA (Fig. 4a). However there was more obvious clade formation in Alberta amongst the RSV type-B sequences that most likely originated from China, South Korea and Vietnam (Fig. 4b).

The structure of the international trees in Fig. 4 speak to what is largely a foreign introduction of new infections however there is evidence of local adaptation of RSV type-A and type-B. In some of the groups of RSV type-A Alberta sequences highlighted in red in Fig. 4a there are closely related sequences from both of the 2014–2015 and the 2015–2016 seasons that lie in the same clade. Local adaptation is more evident in the case of RSV type-B in Fig. 4b where the largest clade that orginated from South Korea contains sequences from both seasons. These data suggest that foreign introduced RSV viruses may also undergo local evolution and that RSV may remain in communities between seasons.

### Comparison of viral load in the patient with RSV phylogenetic pedigree

**Figure 5:**
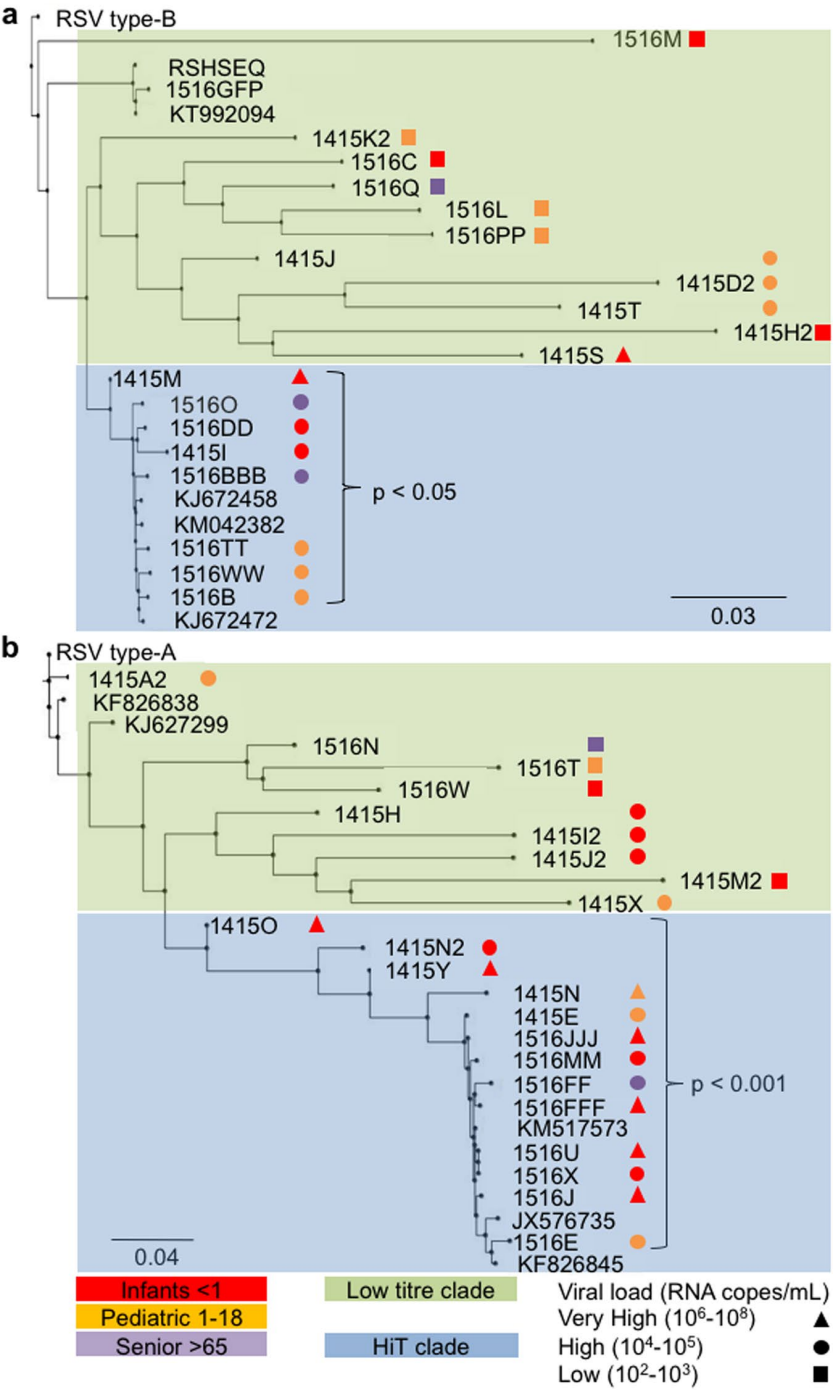
Comparison of RSV phylogenetics with age and patient NP sample titre during the 2014–2015 and 2015–2016 seasons in Alberta Canada. The phylogenetic trees were constructed by a neighbor-joining method using Geneious software from a Geneious alignment plug-in tool. (**a**) RSV type-A in Alberta strains sequenced from 2014–2016. (**b**) RSV type-B in Alberta strains sequenced from 2014–2016. Patient age indicated by red (<1 year), orange (1–18 years) or purple (>65 years). Patient nasopharyngeal sample viral loads indicated by ▲ (10^6^–10^8^ RNA copies/mL), ● (10^4^–10^5^ RNA copies/mL), or ■ (10^2^–10^3^ RNA copies/mL). The green box encompassing the isolates at the top of the tree indicates the resident clades which are lower titre isolates. The blue box encompassing the isolates on the bottom of the figure indicates the HiT clade as described in the results section. P values note the t-test comparison between the viral load of isolates of the resident clades versus the HiT clade.

Patient ages were stratified and viral load data were included in the phylogenetic trees to gain insight into whether age and titre were related to viral pedigree (Fig. 5). We postulated that such associations may lend insight into the variables that drive clade formation. We then added stratified patient ages and viral load data to the phylogenetic trees to gain insight into whether clinical and culture metrics group along with viral relatedness, or may play a role in viral evolution between seasons (Fig. 5). We did not observe an association between the aforementioned clades and patient age groups. However, we did note that the high titre RSV type-A (Fig. 5a) and type-B (Fig. 5b) isolates each tended to group together in clades (in blue), which we termed High Titre (HiT) clades. In contrast, we identified lower titre clades (in green). The patient viral loads in the HiT clades were significantly higher than the titres of infections from resident clades of RSV observed for both RSV type-A (HiT clade mean titre 4.12 × 10^7^ RNA copies/mL ± 1.70 × 10^6^ RNA copies/mL versus a resident mean titre of 3.50 × 10^5^ RNA copies/mL ± 6.53 × 10^5^ RNA copies/mL; p = 0.03) and type-B (HiT clade mean titre 2.57 × 10^6^ RNA copies/mL ± 2.56 × 10^6^ RNA copies/mL versus a resident clade titre of 1.22 × 10^5^ RNA copies/mL ± 2.73 × 10^5^; p = 0.015).

## Discussion

The genetic lineages of RSV types-A and B, and their correlation to patient sample titres, were studied over two seasons that spanned 2014 to 2016 in Alberta, Canada. To the best of our knowledge, this is the first study to compare RSV phylogeny with patient sample titre and endpoints of RSV replication. In doing so, we identified a correlation between the *in vitro* replication kinetics of RSV isolates and patient sample titres. We also observed a preponderance of RSV type-B in those less than one-year of age that was supported by a systematic review and meta-analysis of studies published between 1996 and 2015. Based on these findings, we postulated that those less than one year of age may be a niche host for RSV type-B infections during certain seasons. RSV phylogenetics, combined with patient sample titre analysis, demonstrated that some RSV type-A and B clades could be differentiated based on patient sample titre.

The viruses with the largest sample titre, and foci of infection size, clustered in both RSV type-A and type-B HiT clades. The structures of the phylogenetic trees in our study are similar to what has been reported previously in birth cohorts in Kenya^18^, increasing our confidence in our observations. However, the viruses studied in the Kenyan birth cohort didn’t necessarily cause disease, as most were subclinical infections, whereas our study examined viruses associated with a patient seeking medical attention. Our study therefore will bias for infections that are higher titre as opposed to subclinical infections. RSV genotype can be linked to virulence is supported by a previous study that describes a relationship between RSV Glycoprotein gene variation and disease severity^40^. Altogether, these results suggest that high titre, and likely highly virulent, strains of RSV could be predicted in future seasons based on the highest titre patient isolates from the current season.

We suggest that different selective pressures applied by a niche for each RSV type prevents the genetic convergence of RSV types-A and B. The postulate of niches for each RSV type is supported by the higher prevalence of RSV type-B in patients less than 1 year of age in our patient sample set and meta-analysis. Consistent with a niche hypothesis for RSV types, those less than one-year of age with the highest titre RSV type-B infections in the 2014–2015 season appeared to seed the HiT clade in the 2015–2016 season. Otherwise, it is not known how RSV type-A and RSV type-B remain antigenically distinct since RSV can undergo convergent evolution, particularly in the RSV-G glycoprotein gene segment^41^, which antigenically defines the two types of RSV. There are many factors of RSV biology that should drive the assimilation of the two RSV types: type-A and type-B have mutated at approximately the same rate since 1997^41^, they infect the same hosts (sometimes simultaneously), and there is no animal reservoir. Differences between RSV type-A and type-B plaque morphologies have even been reported previously but it is still unknown why RSV type-A and type-B would plaque differently. Although Kim *et al*. found no association between plaque morphology, viral load and clinical severity^28^. Therefore, we suspect that the different lifestyles and developmental stages posed by different age groups make them amenable as host niches for the different RSV types.

The viral titres we detected in patient samples were influenced mainly by characteristics inherent to the virus. Although we did not access detailed medical history for each of the patients, previous studies have observed that RSV viral load correlates closely to disease severity^6–10,42^. Since RSV has the potential to be transmitted via aerosol^5^, NP viral load may also have implications in increased viral transmission. This concept could help to explain the formation of RSV HiT clades, since a highly transmissible virus is more likely to infect others by aerosol^5^ and to seed a number of infections with high genetic similarity. Even though the HiT clades were characterized by higher titres, the lower titre resident clade viruses, were generally more infectious (higher infectivity indices). We therefore propose that the lower titre clades may evolve from HiT clades by an equilibrium that favours lower titre infections, associated with less disease, that are more infectious and thus easier to transmit. In summary, the HiT and lower titre clades could represent different modes of transmission whereby the high titre viruses may be transmitted by aerosol^5^ and the lower titre viruses may be transmitted by digital transference from surfaces^43^.

Inferred in this study is that RSV type-B must remain in the community between seasons. This is supported by RSV type-B studies showing that it is maintained by subclinical infections in families^16,17^. Further study is required to understand how RSV remains in a community over the summer that would require sampling the population randomly for the presence of subclinical infections. We were limited by the fact that the samples that we obtained for this study were derived from patients that sought medical attention for their infection.

Our pilot study was limited with a relatively small sample size. Greater sampling is required to observe a larger number of high titre isolate samples to prospectively identify those viruses that are most likely to seed the following season. However, analytical depth is afforded to our study by comparison of virological cell culture, viral load, and phylogenetic metrics not previously included in epidemiological studies of RSV. The concordance of RSV replication kinetics, infectious foci and PCR titres in patient NP samples validated the cross-sectional analysis of RSV qRT-PCR titres with phylogenetics. Therefore, in a study or program with sufficient power our study serves as a model to predict the severity of impending RSV outbreaks using virological and phylogenetic surveillance. This may provide the basis of RSV vaccine and drug surveillance programs.

## Electronic supplementary material

Supplementary figure 1

Supplementary Table 1

Supplementary Table 2

Supplementary Table 3
