# Supplementary material for: A Virological and Phylogenetic Analysis of the Emergence of New Clades of Respiratory Syncytial Virus
Source: Sci Rep. 2017 Sep 25;7:12232. doi: 10.1038/s41598-017-12001-6 (PMC5612963; doi:10.1038/s41598-017-12001-6)
Supplement: Supplementary file 1 — Supplementary figure 1 [file 41598_2017_12001_MOESM1_ESM.pdf]

A Virological and Phylogenetic Analysis of the Emergence of New Clades of Respiratory Syncytial Virus

Farah Elawar[1†], Cameron D. Griffiths[1†], Daniel Zhu[1†], Leanne M. Bilawchuk[1], Lionel D. Jensen[1], Lydia Forss[1], Julian Tang[2], Bart Hazes[1], Steven J. Drews[3,4], and David J. Marchant[1*]

[1]Li Ka Shing Institute of Virology, Department of Medical Microbiology and Immunology, University of Alberta, Edmonton, Alberta, Canada.
[2]Clinical Microbiology, University Hospitals of Leicester NHS Trust, Leicester Royal Infirmary, Leicester, UK; Department of Infection, Inflammation and Immunity, University of Leicester, Leicester, UK.
[3]Alberta Provincial Laboratory for Public Health, University of Alberta, Edmonton, AB, Canada.
[4]Department of Laboratory Medicine and Pathology, University of Alberta, Edmonton, Alberta, Canada.

[†]These authors contributed equally
*Corresponding authour

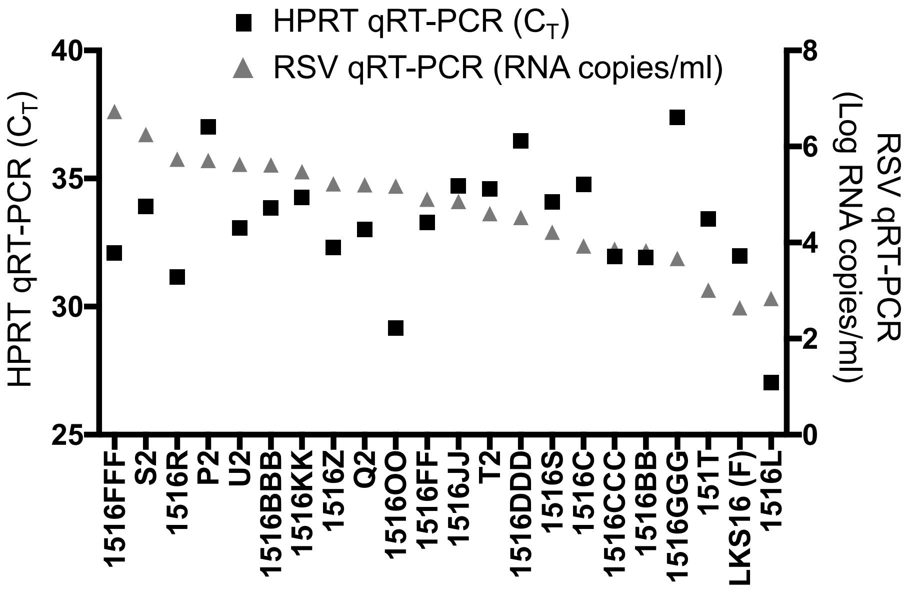

Supplementary Figure 1. Patient HPRT gene mRNA levels in nasopharyngeal swabs. Patient nasopharyngeal swabs were used for viral load determination (denoted by a ▲) corresponding to log FFU/mL on the right axis. HPRT housekeeping gene levels were determined to monitor host patient derived cellular RNA levels. HPRT levels in terms of C_T (denoted by a ▪) on the left axis. Patient samples are ordered from highest to lowest viral load.
